# Impact of Physical Activity and Bio-Psycho-Social Factors on Social Network Addiction and Gender Differences in Spanish Undergraduate Education Students

**DOI:** 10.3390/bs14020110

**Published:** 2024-02-02

**Authors:** Daniel Sanz-Martín, José Luis Ubago-Jiménez, Javier Cachón-Zagalaz, Félix Zurita-Ortega

**Affiliations:** 1 Faculty of Humanities and Social Sciences, Universidad Isabel I, 09003 Burgos, Spain; daniel.sanz6718@ui1.es; 2 Musical, Plastic and Corporal Expression Didactics Department, University of Jaén, 23071 Jaén, Spain; jcachon@ujaen.es; 3 Department of Didactics Musical, Plastic and Corporal Expression, Faculty of Education Science, University of Granada, 18071 Granada, Spain; felixzo@ugr.es

**Keywords:** emotional attention, emotional clarity, emotional repair, sitting time, physical activity, body mass index

## Abstract

Social network use has increased in recent years. Social networks are fast-changing and may cause negative effects such as dependence and addiction. Hence, it was decided to establish two research aims: (1) to identify the social network used by university students and their use levels according to their sex and (2) to analyse how age, body mass index, physical activity, emotional intelligence and social network type affect addiction to social networks according to young people’s sex. A cross-sectional study was designed involving Spanish university students from Education Degrees. The mean age of the participants was 20.84 years (±2.90). Females made up 69.8% of the sample and males 30.2%. An online questionnaire was administered that included sociodemographic questions, IPAQ-SF and TMMS-24. This study found that all students use WhatsApp and more than 97% have YouTube and Instagram accounts. The linear regression model obtained was as follows: social network addiction = 3.355 + 0.336*emotional attention − 0.263*emotional clarity. There is a positive relationship between social network addiction and emotional attention (r = 0.25; *p* < 0.001) and negative relationships between social network addiction and emotional clarity (r = −0.16; *p* = 0.002) and between social network addiction and age (r = −0.17; *p* = 0.001). University students report lower levels of social network addiction and slightly higher levels of social network addiction among females. In addition, there are significant differences between the average social network addiction scores of university students in terms of their use of Telegram, TikTok and Twitch.

## 1. Introduction

Social networks are ‘places in the Internet where people post and share personal and professional information with other persons, known and unknown people’ [1]. Nowadays, three types of social networks exist: general social networks (e.g., Facebook), professional networks (e.g., LinkedIn) and specialised networks (e.g., nVivo) [1]. The most widely used social networks are Facebook, Telegram, Instagram, TikTok, Pinterest, Telegram, Twitter/X, WhatsApp, Snapchat, YouTube, Discord and Twitch [2,3,4].

In addition, social network use levels differ according to individuals’ age, sex (higher in men) and residence locations, and are short-term changeable [2,3]. Furthermore, the use of social networks has increased in recent decades [5], and it is becoming the most widely used tool for communicating and sharing information [6]. At present, 60% of the world’s population (4.80 billion people) is a user of social network, and the average daily use time is 1 h and 24 min [2]. These levels are more striking in Spain, with young people aged 15–26 years spending an estimated 5 h and 30 min on social networks, while young people in the 23–26 years age group spend time at higher levels (5 h and 42 min per day) [7].

Social network use can be beneficial, due to the dissemination of scientific knowledge [8]. However, excessive use can be harmful, as it is associated with low work performances [9], sleep problems [10], depression, anxiety and psychological discomfort [11]. The levels of social network use and its potential harmful effects are so high in terms of levels of social network addiction that there is widespread social concern to avoid the addiction [12]. Addiction is understood as a strong relationship of dependence in which a person is completely committed to a particular situation [13]. Cheng et al. [14] showed that, in spite of this social concern, 8% of the population in northern and western European countries are addicted to social networks, as are 20% of the population of eastern and southern Europe.

Despite the limited evidence in the scientific literature on the relationship between addiction and emotional intelligence [15], emotional intelligence appears to be one of the determinants of social network addiction [16]. As shown by Hormes et al. [17], Olimpia [18], Jarrar et al. [19] and Ali et al. [20], overall emotional intelligence is negatively related to levels of excessive social network use. In contrast, Rivera-Véliz and Araujo-Robles [21] found that the relationships between emotional intelligence and social network addiction may vary depending on the dimensions of emotional intelligence. Specifically, they showed positive relationships between emotional attention and lack of control in social network use and between emotional attention and excessive use, and negative relationships between emotional clarity and social network obsession and between emotional repair and social network obsession [21].

Physical activity could be another potential determinant of addiction, although there is no unanimity among researchers to support it. Kim et al. [22] found no significant relationship between internet addiction and physical inactivity. Mak [23] concluded that there is a positive relationship between social networking and physical activity, but not significantly so. On the other hand, Brailovskaia and Margraf [24] and Selçuk, Lok and Lok [25] showed a negative and significant relationship between physical activity and social network addiction.

Another potential influence on social network addiction could be body mass index, which has a positive relationship. Ali et al. [20] found a positive relationship between body mass index and internet and Facebook addiction in Spanish women suffering from Anorexia Nervosa and other eating disorders. Al Saud et al. [26] also demonstrated a positive relationship between social network addiction and body mass index in Saudi Arabian university students. Ayyıldız and Şahin [27] have also shown a positive relationship between both variables in university students during the COVID-19 pandemic period.

Several studies have measured the social network use levels of Facebook [28], Twitter [29], Instagram [30], Snapchat [31], YouTube [32] and TikTok [33], and there are few studies that have examined relationships between these social network and personal factors [34].

In conclusion, due to the high levels of social network use [2,3], especially in recent decades [5,7] and in the educational environment [35], the health problems associated with the excessive use of social networks [9,10,11], the influence of emotional intelligence on the treatment of addictions [15] and the possibility that dimensions of emotional intelligence affect the levels of social network addiction in different ways [21], it is considered essential to identify the levels and determinants of social network use and addiction among Spanish university students from Education Degrees. Therefore, it would be possible to contribute toward the development of a scientific field that has been under-studied and to design specific initiatives aimed to reverse possible addictions to social networks among future teachers, improving their training and counselling requirements [36] and avoiding these problems being passed on to the next generation. Similarly, it would be important to explore in more detail how the different dimensions of emotional intelligence influence addiction to social networks in order to develop more effective preventive and therapeutic proposals for addiction in order to minimise adverse health effects such as depression and anxiety [11,37].

## 2. Aims and Hypothesis

The research was designed to achieve the following aims:

(1) To identify which social networks are used by university students and their use levels according to sex.
(2) To analyse how age, body mass index, physical activity, emotional intelligence and social network type factors influence social network addiction according to young people’s sex.

Based on these aims, three hypotheses were established:

**H1.**  *Most of the university students show high use levels and lower addiction levels to social networks, with these being higher in men than in women.*

**H2.**  *Age and emotional intelligence are two of the most influential determinants of social network addiction.*

**H3.**  *There are valid predictor models of social network addiction that differ according to sex and addiction dimensions.*

**H4.**  *Social network addiction levels differ according to the use of each social network site.*

## 3. Materials and Methods

### 3.1. Design and Subjects

A cross-sectional descriptive correlational design was used for this study on the social network use levels of Spanish Education undergraduate students and the bio-psycho-social factors conditioning them. This research followed the ethical principles of the Declaration of Helsinki and was approved by the Ethics Committee of the University of Granada (3132/CEIH/2023).

The number of university students in Spain during the academic year 2022–2023 was 150,565 [38]. In order to cover the whole targeted population, the Heads of the Spanish Universities offering Education Degrees were notified about this study and encouraged to send students the questionnaire link.

After the expiration of the response period, 423 students had completed it, but nine of these were excluded due to being outliers during the result screening stage. The final sample (414 students) was representative, with a variance of 95% and a standard deviation of 50, and an estimation error of 4.81%.

The average sample age was 20.84 years (±2.90); 69.8% of the participants were female (*n* = 289) and 30.2% were male (*n* = 125). A total of 44.23% of the Spanish cities participated in the research (23/52) and 64.71% of the Spanish autonomic communities (11/17). A total of 14.5% of students were studying an Early Childhood Education Degree (*n* = 60), 1% Social Education (*n* = 4), 83.3% Primary Education Degree (*n* = 345) and 1.2% were studying a double degree (*n* = 5).

### 3.2. Instrument and Variables

A questionnaire created with the Google^®^ Forms platform (Mountain View, CA, USA) was used throughout the investigation. During the months of May and June 2023, a link to the questionnaire was sent to university students via e-mail by university officials. The questionnaire had the following sections.

#### 3.2.1. Sociodemographic

The section was designed in an ad hoc style and included questions about participants’ sex (male/female), age (years), Spanish residence cities, weight (kilograms) and height (centimetres). Afterward, the body mass index was calculated based on the World Health Organization’s definition [39], dividing the weight expressed in kilograms by height expressed in metres squared.

This section also included questions about the use of different social network sites: Facebook, Snapchat, Telegram, X, Pinterest, LinkedIn, TikTok, Instagram, Twitch, YouTube and WhatsApp. Participants could also include other social network sites. Every social network site was considered a variable, and seven categories were established: daily use, 4–6 days a week, 2–3 days a week, one day a week, sporadic use, do not remember when last used, and do not have the social network site.

#### 3.2.2. Physical Activity

The Spanish version of the IPAQ-SF questionnaire validated by Rodríguez-Muñoz et al. [40] was used. This instrument has been previously used with Spanish university students [41,42].

IPAQ-SF is composed of seven items. The first two items of the questionnaire are related to the number of days and average daily time of vigorous physical activity. The third and fourth items are similar to the first two items, except for moderate physical activity. The fifth and sixth items are similar to the previous ones but related to walking time. The seventh item asks about the average daily time spent sitting on a working day.

Three physical activity variables were used in this research: sitting time (minutes/day), vigorous physical activity (minutes/week) and moderate physical activity (minutes/week).

#### 3.2.3. Emotional Intelligence

The Spanish version of the Trait Meta-Mood Scale (TMMS-24) validated by Fernández-Berrocal et al. [43] for adults aged from 18 to 57 years was included in this section. TMMS-24 has been previously used by Carbonero-Martín et al. [44] and Redondo-Rodríguez et al. [45].

The scale includes three emotional intelligence dimensions: emotional attention, emotional clarity and emotional repair. Each dimension is composed of eight items. Each question is Likert-scaled 1–5, with 1 being “strongly disagree” and 5 “strongly agree”. Each dimension is calculated as a total score of the eight items that compose it, allocating a point for each value on the Likert scale. This means that emotional intelligence is high if the emotional intelligence score increases.

In this study, three variables were established, one per scale dimension, so the final score ranged from 8 to 40 points. Strong internal consistencies were obtained in the three dimensions (emotional attention: α = 0.865; emotional clarity: α = 0.911; emotional repair: α = 0.848).

#### 3.2.4. Social Network Addiction

The Social Network Addiction Scale SNAddS-6S, designed and validated for Spanish adults by Cuadrado et al. [46], was used in this section. SNAddS-6S has been previously used by Cuadrado et al. [47], López-Gil et al. [48] and Gallegos and Flores [49]. Cuadrado et al. [46] found it useful for assessing how addicted people are to social networks and for analysing predictors of social network addiction.

The Social Network Addiction Scale includes 18 items covering five factors: time management (items 1–6), mood modification (items 7–9), relapse (items 10–12), withdrawal (items 13–15) and conflict (items 16–18). The Likert scale responses are 1–5, with 1 being “never” and 5 being “very often”. Each factor is calculated as an average score of the items within it, whereby the average score ranges from 1 to 5. Therefore, it means that social network addiction is high when the value of social network addiction increases.

Six variables related to social network addiction were considered in this investigation: one per scale factor and one per scale average score. Strong internal consistency was obtained for each dimension (time management: α = 0.826; mood modification: α = 0.910; relapse: α = 0.805; withdrawal: α = 0.865; conflict: α = 0.773) and for the average scale score (α = 0.910).

### 3.3. Data Analysis

A statistical analysis was performed using IBM SPSS 26.0 software (International Business Machines Corporation, Armonk, NY, USA). Firstly, a data matrix was generated based on the Google^®^ Forms questionnaire results. Afterward, the data matrix was refined by excluding participants whose z-scores were outside the ±3 range, in order to avoid distorting the statistical analysis [50].

The statistical analysis was conducted in two phases: (1) a descriptive phase of the use levels of social networks and their potential bio-psycho-social factors and (2) a predictive phase of social network addiction levels and correlations with bio-psycho-social factors.

During the first phase, average values and standard deviation were calculated for age, body mass index, three physical activity variables, three emotional intelligence variables and six social network addiction variables. The values were also calculated according to participants’ sex. Afterwards, z-scores were calculated for all variables, and a Student’s *t*-test for independent samples was applied to find out differences in the average values of variables according to students’ sex. In addition, Cohen’s d statistic was calculated to determine effect size, which was considered small if it was close to 0.2, moderate if it was close to 0.5 and large if it was around 0.8 [51].

In the second phase, participants’ percentages using each social network according to the seven categories were calculated. Percentages were calculated for all university students and according to sex. In addition, Chi-square and Cramer’s V test were applied to identify any sex differences in social network use among the students.

In the second statistical phase, multiple linear regression models were developed in order to predict social network addiction levels as a function of bio-psycho-social factors. Therefore, the dependent variable in each model was social network addiction, and the independent variables included in all models were: age, body mass index, sitting time, vigorous physical activity, moderate physical activity, emotional attention, emotional clarity and emotional repair. Furthermore, the analysis of the regression models used the stepwise method, which means that only the significant predictor variables of each model are included, which may differ from one model to another. The models were tested to ensure the following criteria: (1) linearity between the dependent and independent variable before applying the model (using Pearson correlation analysis), (2) independence of model residuals (using the Durbin–Watson statistic), (3) homoscedasticity (using the scatter plot between the model prediction and the residual errors), (4) a normal distribution of errors (using the Kolmogorov–Smirnov test) and (5) non-multicollinearity between independent variables (VIF statistic). In addition, an ANOVA test was used to compare the average scores of social network addiction according to the use categories of each social network.

In all statistical tests, the significance level was set at *p* < 0.05.

## 4. Results

The average age of males was 21.03 years (±2.26), and females, 20.76 years (±3.17). Furthermore, university students’ body mass index was 22.63 (±2.99), which was significantly higher in men than in women (*p* < 0.001, d = 0.62). Time spent performing intense physical activity was significantly higher (*p* < 0.001, d = 0.75) in males than females (399.81 ± 319.81 min/week and 199.47 ± 304.46 min/week, respectively). The women’s average emotional attention score was significantly higher (*p* < 0.001, d = 0.53) than that of men (31.27 ± 5.88 and 28.19 ± 5.94, respectively). Likewise, there were significant differences (*p* < 0.001, d = 0.40) in the emotional repair average score, being higher in males (30.04 ± 5.43) compared to females (27.76 ± 6.08). University students’ average score for social network addiction was 2.18 (±0.75), being slightly higher in women, but without significant difference (*p* = 0.10, d = 0.17). Table 1 shows descriptive statistics for continuous research variables according to participants’ sex, as well as z-score differences.

Fourteen social networks were regularly used by university students. Social network use varied according to students’ sex, with significant differences in eight of them: Telegram (x^2^ = 32.81, *p* < 0.001; V = 0.28), Pinterest (x^2^ = 103.13, *p* < 0.001; V = 0.50), TikTok (x^2^ = 20.39, *p* < 0.01; V = 0.22), Instagram (x^2^ = 14.73, *p* < 0.05; V = 0.19), Twitch (x^2^ = 78.24, *p* < 0.001; V = 0.44), YouTube (x^2^ = 46.77, *p* < 0.001; V = 0.34), BeReal (x^2^ = 10.02, *p* < 0.05; V = 0.16) and Discord (x^2^ = 10.93, *p* < 0.05; V = 0.16). All university students used WhatsApp, and only 0.2% occasionally used Strava. In addition, more than 97% of university students had YouTube and Instagram accounts. Other descriptive statistics of social network use levels are shown in Table 2.

Table 3 presents the multiple linear regression model statistics for predicting social network addiction for the whole participant sample. For the average social network addiction score, the regression model accounts for 12.1% of variance. A positive relationship was observed between social network addiction and emotional attention (r = 0.25; *p* < 0.001), so it can be predicted that every one-point increase in emotional attention is related to a 0.25-point increase in social network addiction. Likewise, negative relationships were observed between social network addiction and emotional clarity (r = −0.16; *p* = 0.002) and between social network addiction and age (r = −0.17; *p* = 0.001). The final model obtained was social network addiction = 3.355 + 0.336*emotional attention − 0.263*emotional clarity.

The regression model related to time management social network addiction is composed of four predictors and explains 10.1% of the variance. In this instance, the model formula is time management social network addiction = 9.974 + 0.320*emotional attention − 0.202*emotional clarity + 0.102*body mass index. Furthermore, there are significant correlations between the score on this dimension of social network addiction and emotional attention (r = 0.24, *p* < 0.001) and emotional clarity (r = −0.11, *p* = 0.03).

The model predicting mood modification social network addiction accounts for 13% of variance and its formula is −1.649 + 0.049*emotional attention − 0.177*emotional clarity—0.136*emotional repair. There are significant correlations between the mood modification social network addiction and emotional attention (r = 0.27, *p* < 0.001) and emotional clarity (r = −0.12, *p* = 0.01).

To predict social network addiction relapse, a model composed of four predictors was found to explain 9.2% of variance. Its formula is as follows: 1.999 + 0.276*emotional attention − 0.250*emotional clarity + 0.107*vigorous physical activity. Relapse social network addiction is related to emotional attention (r = 0.19, *p* < 0.001) and emotional clarity (r = −0.15, *p* = 0.002).

The regression model of the withdrawal social network addiction explains 2.1% of the variance and is −7.685 + 0.143*emotional attention − 0.133*emotional clarity. Also, withdrawal social network addiction is significantly related to emotional attention (r = 0.10, *p* = 0.04) and not to emotional clarity (r = −0.09, *p* = 0.08).

Moreover, the variance explained by the regression model related to conflict social network addiction is 6.2%, being adjusted to 5.7% according to predictor numbers. According to this model, conflict social network addiction = −2.104–0.219*emotional clarity + 0.207*emotional attention. Conflict social network addiction is related to emotional attention (r = 0.14, *p* = 0.01) and emotional clarity (r = −0.16, *p* = 0.02).

Table 4 presents the statistical results of the multiple linear regression models for social network addiction prediction for men. The regression model for the average social network addiction score explains 25.2% of the variance. There is a positive relationship between social network addiction and emotional attention (r = 0.28; *p* = 0.02), so it can be predicted that an increase of a point in emotional attention is related to an increase of 0.28 points in social network addiction. Similarly, there is also a positive relationship between social network addiction and sitting time (r = 0.20; *p* = 0.02). In contrast, a negative relationship was observed between social network addiction and emotional clarity (r = −0.25; *p* = 0.01). The final model obtained was social network addiction = −5.811 + 0.401*emotional attention − 0.368*emotional clarity + 0.225*sitting time −0.199*age.

The model predicting time management social network addiction explains 21% of the variance. There is a significant relationship between males’ time management and emotional attention (r = 0.28; *p* < 0.01) and sitting time (r = 0.22; *p* = 0.03).

The model for mood modification social network addiction accounts for 24.8% of the variance. Mood modification social network addiction has significant relationships with sitting time (r = 0.18; *p* = 0.04), vigorous physical activity (r = −0.198; *p* = 0.03), emotional attention (r = 0.31; *p* < 0.001) and emotional clarity (r = −0.22; *p* = 0.01).

The regression model for relapse social network addiction explains 11.4% of the variance. Relapse social network addiction is positively related to emotional attention (r = 0.24; *p* = 0.01) and negatively related to emotional clarity (r = −0.19; *p* = 0.03).

In relation to withdrawal social network addiction model, the β value of the constant is −7.851 and serves to predict 6.3% of the variance. The relapse social network addiction score is related to age (r = −0.18; *p* = 0.04) and to emotional clarity (r = −0.19; *p* = 0.04).

Finally, regression model-justified variance related to conflict social network addiction is 14.1%, and its formula is conflict social network addiction = −5.321–0.294*emotional clarity + 0.301*emotional attention + 0.188*sitting time. Conflict social network addiction is related to sitting time (r = 0.19; *p* = 0.04), emotional attention (r = 0.20; *p* = 0.03) and emotional clarity (r = −0.23; *p* = 0.01).

Table 5 shows the statistics of the regression model predictors of social network addiction in female university students. The regression model of social network addiction explains 12.4% and includes four predictors: emotional attention, emotional clarity, age and vigorous physical activity. Likewise, social network addiction is related to age (r = −0.17; *p* = 0.03), emotional attention (r = 0.22; *p* < 0.01) and vigorous physical activity (r = 0.15; *p* = 0.01).

The regression model for time management social network addiction includes four predictors: emotional attention, age, emotional clarity and body mass index. The model predicts 10.9% of the variance, and the constant is −8.034. In addition, there are correlations between time management social network addiction and age (r = −0.18; *p* = 0.002), emotional attention (r = 0.21; *p* < 0.001) and vigorous physical activity (r = 0.13; *p* = 0.03).

The mood modification social network addiction is positively associated with emotional attention (r = 0.23; *p* < 0.001) and negatively associated with age (r = −0.13; *p* = 0.03). The model obtained to predict mood modification social network addiction represents 9.9% of the variance and includes four predictors (emotional attention, emotional clarity, age and emotional repair).

The final model obtained for relapse social network addiction in women was −1.153 + 0.154*vigorous physical activity + 0.232*emotional attention − 0.219*emotional clarity. This model predicts 4% of the variance. Relapse social network addiction is positively related to emotional attention (r = 0.16; *p* = 0.01) and vigorous physical activity (r = 0.17; *p* = 0.04) and negatively related to emotional clarity (r = −0.13; *p* = 0.03).

The withdrawal social network addiction model predicts 4%, and its formula is 4.170 − 0.181*age + 0.125*emotional attention. There is a positive relationship between withdrawal social network addiction and emotional attention (r = 0.12; *p* = 0.04) and a negative relationship with age (r = −0.18; *p* = 0.03).

Finally, conflict social network addiction predicts 4% of the variance, has a constant of 2.793 and includes emotional clarity and emotional attention as predictors. Conflict social network addiction is significantly related to emotional attention (r = 0.12; *p* = 0.04) and emotional clarity (r = −0.12; *p* = 0.04).

The average scores of the social network addiction variables were compared according to categories of social network use, but post hoc tests could not be calculated because there are more than fifty groups. There is a significant difference between the mean social network addiction scores for all participants and Telegram (F (58,413) = 1.447, *p* = 0.02), as well as TikTok (F (58,413) = 1.414, *p* = 0.03) and Twitch (F (58,413) = 1.470, *p* = 0.02). There is also a difference between the average time management social network addiction scores of participants according to TikTok (F (23,413) = 2.285, *p* < 0.001) and Instagram use (F (23,413) = 3.126, *p* < 0.001). Likewise, there is a difference in averages regarding mood modification social network addiction and TikTok (F (12,413) = 1.993, *p* = 0.02).

It was found that there is no significant difference between average male social network addiction and use of all social networks. There is a significant difference between time management social network addiction in males and the use of TikTok (F (21,124) = 1.838, *p* = 0.02), WhatsApp (F (21,124) = 22.287, *p* < 0001), BeReal (F (21,124) = 2.071, *p* = 0.01) and Discord (F (21,124) = 1.785, *p* = 0.03). A significant difference was found between mood modification social network addiction in men and Pinterest use (F (12,124) = 2.346, *p* = 0.01). Comparing the Facebook addiction category averages, they are statistically significant in relapse social network addiction (F (11,124) = 1.971, *p* = 0.04) and withdrawal social network addiction (F (10,124) = 2.171, *p* = 0.02). There are also differences in the conflict social network addiction scores between the categories of Telegram (F (11,124) = 2.125, *p* = 0.02), Pinterest (F (11,124) = 2.258, *p* = 0.02) and BeReal use (F (11,124) = 2.111, *p* = 0.03).

Regarding a comparison of women’s total social network addiction scores according to the categories of social network use, significant differences were obtained for LinkedIn (F (56,288) = 1.446, *p* = 0.03) and Twitch (F (56,124) = 1.449, *p* = 0.03). Furthermore, differences in relapse social network addiction were also obtained for LinkedIn (F (12,288) = 1.923, *p* = 0.03) and Twitch (F (12,288) = 1.806, *p* = 0.04). Women’s scores on time management social network addiction differed as a function of Instagram use (F (23,288) = 2.650, *p* < 0.001), just like for withdrawal social network addiction and LinkedIn (F (12,288) = 1.886, *p* = 0.04), conflict social network addiction and Pinterest (F (12,288) = 1.869, *p* = 0.04) and conflict social network addiction and TikTok (F (12,288) = 1.808, *p* = 0.05).

## 5. Discussion

The aims of this research were (1) to identify which social networks are used by university students and their use levels according to sex and (2) to analyse the influences of age, body mass index, physical activity, emotional intelligence and type of social network as factors on addiction to social networks according to young people’s sex.

WhatsApp is the most used social network by Spanish university students from Education Degrees, as all of them use it, and more than 98% of them do so on a daily routine. Instagram is the second-most used social network, with 80.8% of men and 91.7% of women using it daily. In contrast, Strava is ranked as the least used social network, with 0.3% of women using it sporadically, and none of the rest using it at all. These results are similar to the results obtained by Martin and Medina [52] who obtained Instagram and WhatsApp as the most used social networks, but in inverse order to the Spanish university students in this study. On the other hand, these results are different from those found by Chaffey [2], who showed that Facebook and YouTube were the most used.

Social network use varies according to university students’ sex, with significant differences in Telegram, Pinterest, TikTok, Instagram, Twitch, YouTube, BeReal and Discord use. Men use Facebook, Snapchat, X, Pinterest LinkedIn, TikTok, Instagram, BeReal and Strava less than women. In contrast, men use Telegram, Twitch, YouTube and Discord more. Forner [53] showed that Facebook, YouTube, Instagram and TikTok are more used by Spanish women than by men. Sex differences in social network use may be due to social network purposes, as women may prefer to use them to share videos and photos and to search for fashion items, which are activities more associated with Facebook, Instagram and TikTok [53,54].

Levels of social network addiction among university students are not worrying, reaching a mean score of 2.18 (±0.75) on a range of 1–5 points. Higher scores were obtained in time management social network addiction and lower scores in the conflict dimension. Although there were no significant differences according to sex, women obtained higher addiction scores in the time management, mood modification, relapse and withdrawal dimensions, and men in the conflict dimension.

Students’ average body mass index was 22.63, which is considered normal according to World Health Organization [39] recommendations. This value is lower than the value obtained by Rodríguez-Besteiro et al. [55] for Spanish university students (23.65 ± 2.93). In addition, men’s body mass index was significantly higher than women’s in this study. The positive and non-significant relationship between body mass index and social network addiction could be partially due to social network use being considered a low-intensity physical activity [56], which is associated with low energy expenditure [57] and therefore a greater likelihood of weight gain [58]. This would be increased because addiction involves more social networking time [59].

Males perform more vigorous physical activity than females (399.81 ± 319.81 min/week vs. 199.47 ± 304.46 min/week), with a significant difference and being twice as long per week. The average time spent in moderate vigorous physical activity is much higher than World Health Organization [56] recommendations, as they perform 404.26 min/week and at least 150 min/week is recommended. Corella et al. [60], Rodriguez-Larrad et al. [61] and Sañudo et al. [62] obtained higher levels of moderate vigorous physical activity. In contrast, the physical activity levels observed by Romero-Blanco et al. [63] were lower.

Multiple linear regression models were obtained for all male and female participants according to the total social network addiction score and all its dimensions, which were valid and different for all participants. The regression models obtained for social network addiction, time management social network addiction and mood modification social network addiction for men are particularly relevant, as they predict more than 21% of the variance. The variables most predictive of social network addiction are emotional attention and emotional clarity, as they are found in most models. In addition, a positive relationship between emotional attention and social network addiction was found to predominate, and a less strong and negative relationship between emotional clarity and social network addiction was observed. These results serve to further explore the influence of emotional intelligence on social network addiction. This is because previous research [17,18,19,20,64] found a negative relationship between this addiction and emotional intelligence measured in general, but based on the results obtained in this study, this relationship is different depending on the dimensions of emotional intelligence. This finding would imply that in order to reduce the level of addiction to social networks among Spanish students from Education Degrees, it would be important to design intervention proposals based on improving emotional clarity, although this might not be useful for students of other university degrees, such as Science, because their levels of emotional intelligence are lower [65]. Similarly, this difference could be due to the socio-emotional training that teachers receive in order to cope with future daily interactions with their pupils [66].

Likewise, vigorous physical activity, sitting time and age also have an influence in some regression models, with their predictions being positive for the first two variables and negative for age. The results obtained in this research provide relevant and novel findings to the scientific literature, as few previous studies have examined the relationships between social network use and personal factors [34]. In contrast to the results of the present study, previous research has found a negative relationship between physical activity and social network addiction [24,25]. Furthermore, the type of physical activity that predicts social network addiction differs according to the gender of the Spanish university students, with sedentary time standing out as a predictor for men and vigorous physical activity for women. This sex difference may be due to the fact that men use social networks to play video games [67], which is a sedentary activity, and women use them to share videos and photos [53], which could be physical activity.

However, the regression models obtained are valid, although there is a high percentage of variance in social network addiction remaining unexplained, so there could be other predictors, such as subjective well-being [68], emotional exhaustion, cynicism [69] and place of residence while in school (hostel, home and college) [70].

There are significant differences between social network addiction average scores based on Telegram use. In addition, these differences are significant in LinkedIn and Twitch use for women. The levels of relapse and withdrawal social network addiction for men differ as a function of Facebook use. These results cannot be discussed in terms of the scientific literature because no similar studies have been found, but it seems to suggest that more social network use increases the likelihood of addiction, as evidenced by Cudo et al. [28] with Facebook minutes of use.

The study has several limitations. Firstly, it is a cross-sectional study carried out in June, meaning responses relate to a specific period in which students have a heavier academic load. Although this limitation is common to all cross-sectional studies, we have tried to compensate by detailing all characteristics of the investigation. An additional limitation is a consequence of participant selection. All Spanish university students from Education Degrees were asked to receive links to questionnaires through their university heads, who decided if they would forward them. Thus, no specific selection was carried out, and it was decided to include all participants who answered the questionnaire correctly. This limitation was compensated by a nationally representative sample covering most Spanish areas. Finally, there is a third limitation intrinsic to the physical activity questionnaire used. Even though this instrument has been validated for Spanish adults and has been used in numerous previous investigations, there are other more accurate instruments, such as accelerometers. It was decided to use a questionnaire because it allowed information to be collected from many participants from different Spanish areas in a short period of time and at no economic cost.

The study results allow us to establish future lines of research. Firstly, it would be useful to carry out similar studies that include more habits, such as alcohol consumption, smoking and other harmful substances. This would provide a more comprehensive picture of the type of healthy lifestyle that university students lead. Secondly, a longitudinal study should be considered to find out how healthy habits change over time and how they are influenced by the use of social networks. And finally, it would be appropriate to reduce students’ sitting time, as they are spending an average of almost four hours a day of sedentary behaviour. In order to reverse these levels, physical activity promotion programmes could be designed, such as daily university sport tournaments, active breaks during classes [71] or extra-curricular training activities, as they are an effective means of acquiring and consolidating healthy lifestyle habits among Spanish university students [72]. Actions could also be designed to improve emotional intelligence levels, although these are not excessively low. These interventions should focus on improving emotional clarity and repair, especially for women. This would provide several benefits, such as lowering social network addiction levels [16,18]. Likewise, these proposals could be promoted through social networks, especially WhatsApp and Instagram, as they are the most used networks by students.

## 6. Conclusions

In this investigation, four hypotheses were confirmed:

**H1.**  *Most of the university students show high use levels and lower addiction levels to social network, being these higher in men than in women.*

A high use level has been shown among students because they all have WhatsApp, and more than 98% use it on a daily basis. Likewise, almost 90% of young people use Instagram every day. In addition, more than 69% of university students use TikTok for at least 2–3 days per week.

University students have low levels of social network addiction because their scores are less than half of the maximum scores. These levels are slightly higher in women.

**H2.**  *Age and emotional intelligence are two of the most influential determinants of social network addiction.*

Emotional attention is positively related to social network addiction, while emotional clarity is negatively related to social network addiction.

Age has a negative influence on social network addiction among university students, as well as among women.

**H3.**  *There are valid predictor models of social network addiction which differ according to sex and addiction dimensions.*

This hypothesis was also confirmed. The regression models differ across constants and type and relationship of predictors. In addition, attention and emotional clarity are jointly included in most of the models. The most-predictive models are those concerning total social network addiction, time management social network addiction and mood modification social network addiction for men, as they explain more than 20% of the variance.

**H4.**  *Social network addiction levels differ according to use of each social network site.*

Significant differences were shown among university students’ mean social network addiction scores based on their Telegram, TikTok and Twitch use. Women’s addiction levels also differ according to their use of LinkedIn and Twitch.

## Figures and Tables

**Table 1:** Continuous research variables, descriptive analysis.

	Total (*n* = 414)	Males (*n* = 125)	Females (*n* = 289)	Z-Scores	
				T-Student	Cohen’s d
Age	20.84 (2.90)	21.03 (2.26)	20.76 (3.17)	1.06	0.11
BMI	22.63 (2.99)	23.59 (2.66)	22.20 (3.03)	4.29 ***	0.62
ST-T (min/d)	232.64 (200.74)	236.27 (182.45)	231.05 (208.54)	0.10	0.01
VPA (min/w)	260.57 (322.35)	399.81 (319.81)	199.47 (304.46)	6.76 ***	0.75
MPA (min/w)	143.70 (250.50)	172.74 (233.89)	130.95 (256.80)	1.84	0.20
EA	30.33 (6.06)	28.19 (5.94)	31.27 (5.88)	−5.00 ***	0.53
EC	28.16 (6.34)	28.95 (6.00)	27.82 (6.46)	1.66	0.17
ER	28.45 (5.98)	30.04 (5.43)	27.76 (6.08)	3.69 ***	0.40
TM-SNA	2.58 (0.86)	2.47 (0.77)	2.63 (0.89)	−1.89	0.20
MM-SNA	2.49 (1.08)	2.35 (1.04)	2.57 (1.08)	−1.68	0.20
R-SNA	2.16 (1.05)	2.05 (0.99)	2.20 (1.06)	−1.14	0.13
W-SNA	1.84 (0.97)	1.75 (0.90)	1.87 (0.99)	0.20	0.12
C-SNA	1.83 (0.90)	1.85 (0.92)	1.83 (0.89)	−1.63	0.02
Total SNA	2.18 (0.75)	2.10 (0.73)	2.22 (0.76)	0.10	0.17

Note: Body mass index (BMI); sitting time (ST-T); vigorous physical activity (VPA); moderate physical activity (MPA); emotional attention (EA); emotional clarity (EC); emotional repair (ER); time management social network addiction (TM-SNA); mood modification social network addiction (MM-SNA); relapse social network addiction (R-SNA); withdrawal social network addiction (W-SNA); conflict social network addiction (C-SNA); social network addiction (SNA); minutes (min); week (w); day (d); *p*-value ≤ 0.001 (***).

**Table 2:** Descriptive statistics of social network use levels.

Social Network	Sex	7 Days (%)	4–6 Days (%)	2–3 Days (%)	1 Day (%)	Occasionally (%)	Do Not Remember Last Time (%)	Do Not Have This Social Network (%)	X^2^	V
Facebook	Male	4	1.6	4.8	2.4	8.8	21.6	56.8	4.03	0.11
	Female	4.2	2.4	5.9	1.0	15.2	20.4	50.9		
	Total	4.1	2.2	5.6	1.4	13.3	20.8	52.7		
Snapchat	Male	0.8	0	1.6	1.6	4.8	18.4	72.8	9.63	0.15
	Female	1.4	0.3	2.1	2.1	6.2	30.8	57.1		
	Total	1.2	0.2	1.9	1.9	5.8	27.1	61.8		
Telegram	Male	1.6	1.6	7.2	5.6	30.4	15.2	38.4	32.81 ***	0.28
	Female	2.1	1.0	2.1	1.0	14.2	19.7	59.5		
	Total	1.9	1.2	3.6	2.7	19.1	18.4	53.1		
X	Male	24	5.6	11.2	6.4	13.6	8	31.2	4.89	0.11
	Female	20.1	9.3	10.4	4.8	18.3	10.4	26.6		
	Total	21.3	8.2	10.6	5.3	16.9	9.7	28		
Pinterest	Male	0.8	0.8	4	1.6	7.2	10.4	75.2	103.13 ***	0.50
	Female	4.2	7.6	14.9	6.9	29.4	13.5	23.5		
	Total	3.1	5.6	11.6	5.3	22.7	12.6	39.1		
LinkedIn	Male	0	0	2.4	1.6	9.6	3.2	83.2	9.22	0.15
	Female	0.3	0.3	1.7	1.4	8.7	12.1	75.4		
	Total	0.2	0.2	1.9	1.4	8.9	9.4	77.8		
TikTok	Male	51.2	16.8	3.2	2.4	3.2	3.2	20	20.39 **	0.22
	Female	69.6	6.6	4.2	0.7	3.5	1.0	14.5		
	Total	64	9.7	3.9	1.2	3.4	1.7	16.2		
Instagram	Male	80.8	11.2	2.4	0.8	0.8	1.6	2.4	14.73 *	0.19
	Female	91.7	3.1	1.0	0.3	1.7	0.5	1.4		
	Total	88.4	5.6	1.4	0.5	1.4	1	1.7		
Twitch	Male	12	4.8	16	6.4	23.2	8.8	28.8	78.24 ***	0.44
	Female	2.4	1.4	5.2	1.4	9.3	9.0	71.3		
	Total	5.3	2.4	8.5	2.9	13.5	8.9	58.5		
YouTube	Male	56.8	19.2	12.8	0.8	8.8	0	1.6	46.77 ***	0.34
	Female	27.0	16.3	18.0	7.6	23.9	3.8	3.5		
	Total	36.0	17.1	16.4	5.6	19.3	2.7	2.9		
WhatsApp	Male	98.4	0.8	0.8	0	0	0	0	0.86	0.05
	Female	98.3	1.0	0.3	0	0.3	0	0		
	Total	98.3	1	0.5	0	0.2	0	0		
BeReal	Male	2.4	0	1.6	0	0.8	0	95.2	10.02 *	0.16
	Female	5.9	0.7	0	0	0	0	93.4		
	Total	4.8	0.5	0.5	0	0.2	0	94		
Discord	Male	4	0	0.8	0	0	0	95.2	10.93 *	0.16
	Female	0.3	0	0	0	0.3	0	99.3		
	Total	1.4	0	0.2	0	0.2	0	98.1		
Strava	Male	0	0	0	0	0	0	100	0.43	0.03
	Female	0	0	0	0	0.3	0	99.7		
	Total	0	0	0	0	0.2	0	99.8		

Note: Chi-square test (x^2^); Cramer test (V); *p* ≤ 0.05 (*); *p* ≤ 0.01 (**); *p* ≤ 0.001 (***).

**Table 3:** Regression results for the whole sample.

		β	SE	*p*	VIF	R^2^	Adjusted R^2^	RSE	F	D-W
Total SNA	EA	0.336	0.049	<0.001	1.116	0.125	0.121	0.938	29.419 ***	1.986
	EC	−0.263	0.049	<0.001	1.116					
	Constant	3.355								
TM-SNA	EA	0.320	0.049	<0.001	1.125	0.108	0.101	0.948	16.490 ***	2.025
	EC	−0.202	0.049	<0.001	1.117					
	BMI	0.102	0.047	0.031	1.012					
	Constant	9.974								
MM-SNA	EA	0.348	0.049	<0.001	1.117	0.136	0.130	0.933	21.566 ***	1.891
	EC	−0.177	0.053	<0.001	1.344					
	ER	−0.136	0.051	0.008	1.239					
	Constant	−1.649								
R-SNA	EA	0.276	0.050	<0.001	1.116	0.098	0.092	0.953	14.910 ***	1.960
	EC	−0.250	0.050	<0.001	1.116					
	VPA	0.107	0.047	0.024	1.013					
	Constant	1.999								
W-SNA	EA	0.143	0.051	0.006	1.116	0.026	0.021	0.989	5.431 **	1.921
	EC	−0.133	0.051	0.010	1.116					
	Constant	−7.685								
C-SNA	EC	−0.219	0.050	<0.001	1.116	0.062	0.057	0.971	13.500 ***	1.944
	EA	0.207	0.050	<0.001	1.116					
	Constant	−2.104								

Note: Body mass index (BMI); emotional attention (EA); emotional clarity (EC); emotional repair (ER); vigorous physical activity (VPA); time management social network addiction (TM-SNA); mood modification social network addiction (MM-SNA); relapse social network addiction (R-SNA); withdrawal social network addiction (W-SNA); conflict social network addiction (C-SNA); social network addiction (SNA). ** *p* ≤ 0.05; *** *p* ≤ 0.001.

**Table 4:** Regression results for males.

		β	SE	*p*	VIF	R^2^	Adjusted R^2^	RSE	F	D-W
Total SNA	EA	0.401	0.081	<0.001	1.094	0.276	0.252	0.865	11.457 ***	1.929
	EC	−0.368	0.082	<0.001	1.111					
	ST	0.225	0.078	0.005	1.019					
	Age	−0.199	0.079	0.013	1.024					
	Constant	−5.811								
TM-SNA	EA	0.380	0.083	<0.001	1.094	0.236	0.210	0.889	9.258 ***	1.999
	EC	−0.281	0.084	<0.001	1.111					
	ST	0.246	0.081	0.003	1.019					
	Age	−0.192	0.081	0.019	1.024					
	Constant	−3.404								
MM-SNA	EA	0.430	0.081	<0.001	1.094	0.272	0.248	0.867	11.204 ***	1.965
	EC	−0.350	0.082	<0.001	1.111					
	ST	0.205	0.079	0.010	1.019					
	Age	−0.177	0.079	0.027	1.024					
	Constant	−5.004								
R-SNA	EA	0.316	0.088	<0.001	1.088	0.128	0.114	0.941	8.953 ***	1.911
	EC	−0.281	0.088	0.002	1.088					
	Constant	−7.908								
W-SNA	EA	−0.215	0.088	0.016	1.017	0.078	0.063	0.968	5.189 **	1.665
	EC	−0.210	0.088	0.018	1.017					
	Constant	−7.851								
C-SNA	EC	−0.294	0.087	0.001	1.093	0.162	0.141	0.927	7.772 ***	1.857
	EA	0.301	0.087	0.001	1.093					
	ST	0.188	0.084	0.027	1.013					
	Constant	−5.321								

Note: emotional attention (EA); emotional clarity (EC); sitting time (ST); time management social network addiction (TM-SNA); mood modification social network addiction (MM-SNA); relapse social network addiction (R-SNA); withdrawal social network addiction (W-SNA); conflict social network addiction (C-SNA); social network addiction (SNA). ** *p* ≤ 0.05; *** *p* ≤ 0.001.

**Table 5:** Regression results for females.

		β	SE	*p*	VIF	R^2^	Adjusted R^2^	RSE	F	D-W
Total SNA	EA	0.300	0.0060	<0.001	1.178	0.136	0.124	0.936	11.223 ***	1.976
	EC	−0.209	0.060	0.001	1.185					
	Age	−0.161	0.055	0.004	1.012					
	VPA	0.124	0.055	0.026	1.006					
	Constant	2.644								
TM-SNA	EA	0.285	0.060	<0.001	1.172	0.121	0.109	0.944	9.770 ***	1.963
	Age	−0.191	0.056	0.001	1.027					
	EC	−0.199	0.061	0.015	1.193					
	BMI	0.133	0.056	0.019	1.025					
	Constant	−8.034								
MM-SNA	EA	0.311	0.061	<0.001	1.179	0.112	0.099	0.949	8.913 ***	1.839
	EC	−0.124	0.066	0.042	1.404					
	Age	−0.127	0.056	0.025	1.017					
	ER	−0.131	0.063	0.038	1.255					
	Constant	−2.195								
R-SNA	VPA	0.154	0.057	0.007	1.006	0.092	0.082	0.958	9.616 ***	1.988
	EA	0.232	0.061	<0.001	1.178					
	EC	−0.219	0.061	<0.001	1.172					
	Constant	−1.153								
W-SNA	Age	−0.181	0.058	0.002	1.001	0.047	0.040	0.980	7.046 ***	2.031
	EA	0.125	0.058	0.031	1.001					
	Constant	4.170								
C-SNA	EC	−0.198	0.062	0.002	1.171	0.047	0.040	0.980	7.068 ***	1.941
	EA	0.193	0.062	0.002	1.171					
	Constant	2.793								

Note: Body mass index (BMI); emotional attention (EA); emotional clarity (EC); emotional repair (ER); vigorous physical activity (VPA); time management social network addiction (TM-SNA); mood modification social network addiction (MM-SNA); relapse social network addiction (R-SNA); withdrawal social network addiction (W-SNA); conflict social network addiction (C-SNA); social network addiction (SNA). *** *p* ≤ 0.001.

## Data Availability

The data used to support the findings of the current study are available from the corresponding author upon request.
